# Atractylodin Inhibits Interleukin-6 by Blocking NPM-ALK Activation and MAPKs in HMC-1

**DOI:** 10.3390/molecules21091169

**Published:** 2016-09-02

**Authors:** Hee-Sung Chae, Young-Mi Kim, Young-Won Chin

**Affiliations:** College of Pharmacy and Integrated Research Institute for Drug Development, Dongguk University-Seoul, 32 Dongguk-lo, Ilsandong-gu, Goyang-si, Gyeonggi-do 10326, Korea; chaeheesung83@gmail.com (H.-S.C.); 0210121@hanmail.net (Y.-M.K.)

**Keywords:** atractylodin, *Atractylodes lancea*, interleukin-6, nucleophosmin-anaplastic lymphoma kinase, human mast cell-1

## Abstract

Atractylodin is one of the major constituents of the rhizome of *Atractylodes lancea*, which is widely used in Korean traditional medicine as a remedy for the treatment of gastritis and gastric ulcers. Despite of a major constituent of widely used botanical to treat inflammatory responses little is known about anti-inflammatory effect of atractylodin in the human mast cell (HMC-1). Hence, we evaluated the effect of atractylodin on the release of IL-6, the involvement of nucleophosmin-anaplastic lymphoma kinase (NPM-ALK) and mitogen-activated protein kinases (MAPKs) in phorbol-12-myristate-13-acetate and A23187-induced HMC-1. In addition, Janus kinase 2 (JAK2), signal transducer and activator of transcription 3 (STAT3), phospholipase C (PLC) gamma 1, and AKT phosphorylation relevant to NPM-ALK signal pathway were assessed. IL-6 levels in the HMC-1 stimulated by phorbol-12-myristate-13-acetate and A23187 were apparently decreased by the treatment of atractylodin. Concurrently, atractylodin not only inhibited the phosphorylation of NPM-ALK, but also suppressed the phosphorylation of JAK2, STAT3, PLC gamma 1, and AKT. Furthermore, the activated mitogen-activated protein kinases (MAPKs) by phorbol-12-myristate-13-acetate and A23187 were inhibited by atractylodin. These results suggested that atractylodin might have a potential regulatory effect on inflammatory mediator expression through blockade of both the phosphorylation of MAPKs and the NPM-ALK signaling pathway.

## 1. Introduction

**Figure 1:**
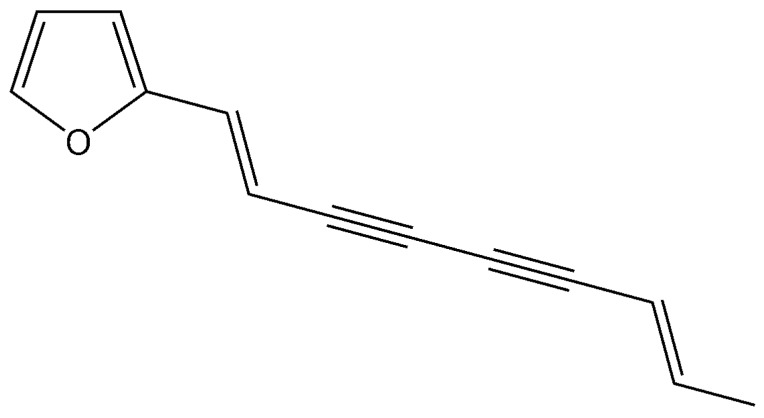
Structure of atractylodin.

*Atractylodes lancea* (Thunb.) DC. (Asteraceae) has been widely used in various Korean traditional medicine prescriptions for the treatment of gastric disorders and gastric ulcers in eastern Asia [1]. It has been reported that *A. lancea* attenuates stomach damage by exerting anti-ulcer effects and inhibiting gastric secretions [2,3]. Previous phytochemical reports of *A. lancea* led to the isolation of essential oils, including polyacetylenes, phenylpropanoids, and dihydrobenzofuran [1,4,5]. The pharmacological investigation on atractylodin (Figure 1), one of the major constituents of *A. lancea*, has been reported only in a few reports such as lipase inhibitory activity [6]. However, its anti-inflammatory effect on the human mast cell-1 (HMC-1) remains unknown.

Anaplastic lymphoma kinase (ALK) is an orphan receptor tyrosine kinase originally identified as part of the nucleophosmin (NPM)-ALK fusion gene in anaplastic large-cell lymphomas having a translocation [7,8,9]. The activated ALK kinase can act as an oncogene in different cell systems and induce malignant transformation of fibroblasts [10]. Investigation of the mode of action of NPM-ALK reveals that NPM-ALK-mediated lymphomagenesis involves JAK2/STAT3 activation, in addition to the activation of AKT and phospholipase C gamma 1 pathways [11,12,13,14]. Moreover, phospholipase C (PLC) is crucial for FcɛRI-mediated mast cell activation. Mast cells function as major effectors in high-affinity IgE receptor (FcɛRI)-dependent allergic reactions [15]. Phorbol 12-myristate 13-acetate (PMA) mimics the function of the second messenger, diacylglycerol (DAG), which is an activator of signaling kinases in the protein kinase C (PKC) pathway [16]. PKC activation is involved in NPM-ALK-mediated mitogenic signaling, which suggests that the inhibition of this activity can interfere with the pathogenic effects of NPM-ALK [17]. ALK positively regulates AKT, JAK/STAT, and PLCγ activation, and phosphorylation of ALK is involved in FcεRI transcription via recruitment to its downstream pathways [18]. NPM-ALK, in turn, induces interleukins through activation of its key signal transmitter, STAT3, leading to a rationale that NPM-ALK might be therapeutic target to modulate interleukins [19,20]. Moreover, ALK fusion genes are associated with both anaplastic large-cell lymphoma and inflammatory myofibroblastic tumors [21].

In this study, we determined whether or not PMA plus A23187 were able to stimulate the NPM-ALK pathway. Additionally, atractylodin was evaluated for its inhibitory effect on IL-6 production and activation of the NPM-ALK and MAPK pathways in PMA/A23187-induced HMC-1 cells.

## 2. Results

### 2.1. The Effects of Atractylodin on PMA Plus A23187-Induced ALK Pathway Activation

**Figure 2:**
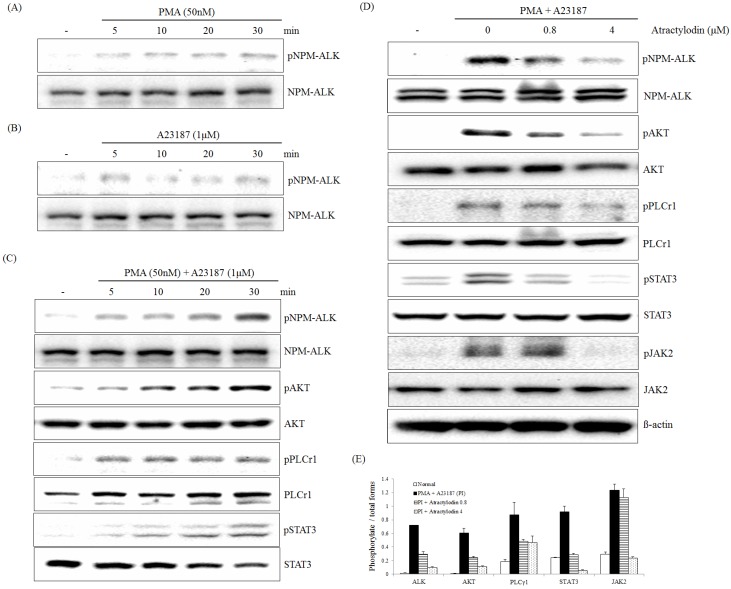
Effect of atractylodin on the PMA plus A23187-induced activation of the NPM-ALK pathway in HMC-1. HMC-1 was treated with PMA at the indicated times for 50 nM and phospho (Tyr646)- and total NPM-ALK were detected by immunoblot analysis (**A**); HMC-1 was treated with A23187 at the indicated times for 1 μM and phospho (Tyr646)- and total NPM-ALK were detected by immunoblot analysis (**B**); HMC-1 was treated with PMA plus A23187 at the indicated times for 50 nM and 1 μM and phospho- and total NPM-ALK, AKT, PLCγ1 and STAT3 were detected by immunoblot analysis (**C**); HMC-1 was treated with the indicated concentrations of atractylodin for 0.5 h prior to being incubated with PMA (50 nM) plus A23187 (1 μM) for 0.5 h and phospho and total NPM-ALK, AKT, PLCγ1, JAK2, and STAT3 were detected by immunoblot analysis as described in Materials and Methods (**D**); The immunoblot signals were quantified using Molecular Analyst/PC densitometry software (Bio-Rad). Densitometric analysis of phosphorylated isoforms is reported (**E**).

The effect of atractylodin on NPM-ALK, JAK2, STAT3, AKT, and PLCγ1 phosphorylation induced by PMA plus A23187 was examined using immunoblot analysis. A cellular phosphorylation assay demonstrated that phosphorylation of NPM-ALK was not significantly activated in either PMA- or A23187-treated HMC-1 cells (Figure 2A,B). However, treatment with PMA plus A23187 led to a time-dependent activation of NPM-ALK tyrosine (646) phosphorylation in HMC-1 cells. Phosphorylated Tyr646 of NPM-ALK (equivalent to Tyr1586 of full-length ALK) is required for its interaction with PLCγ and activation of PLCγ by NPM-ALK is a crucial step for mediating its mitogenic activity, which is important in the pathogenesis of anaplastic lymphomas [22]. ALK activation triggers multiple members of the ALK pathway, including phosphorylated JAK2, STAT3, PLCγ, and AKT [23]. JAK2 phosphorylates adjacent STAT3, but full activation of STAT3 may require other protein modifications, such as serine phosphorylation [24]. Treatment with PMA plus A23187 led to a time-dependent activation of JAK2, STAT3, PLCγ, and AKT phosphorylation in HMC-1 cells (Figure 2C). Treatment of NPM-ALK–activated HMC-1 cells with atractylodin caused a concentration-dependent inhibition of NPM-ALK tyrosine (646) phosphorylation. Inhibition of interleukin signaling through the JAK2-STAT3 pathway in immune cells by atractylodin was observed in this study. Similarly, atractylodin inhibited JAK2, STAT3, PLCγ, and AKT phosphorylation in HMC-1 cells in a concentration-dependent manner with equivalent potency (Figure 2D).

### 2.2. The Effects of Atractylodin on PMA Plus A23187-Induced MAPKs Activation

**Figure 3:**
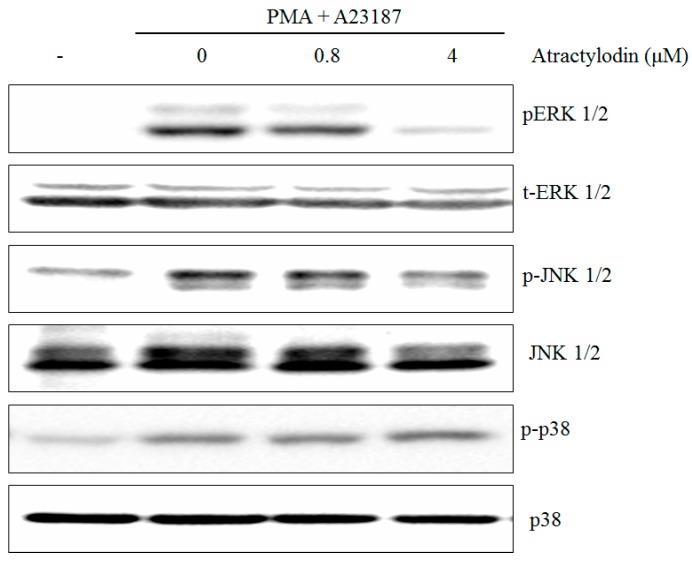
Effects of atractylodin on the phosphorylation of MAPKs in stimulated HMC-1. HMC-1 was treated with the indicated concentrations of atractylodin for 0.5 h prior to being incubated with PMA (50 nM) plus A23187 (1 μM) for 0.5 h. Whole cell lysates were then analyzed by Western blot. Equal amounts of protein (20 μg) were then separated by SDS-polyacrylamide gel electrophoresis and immunoblotted with antibodies.

The stimulation of HMC-1 cells with PMA plus A23187 resulted in an increased phosphorylation of all three types of MAPKs, p38, JNK, ERK, after 0.5 h post-treatment. Atractylodin apparently suppressed phosphorylation of ERK1/2 and JNK1/2, but not the phosphorylation of p38 in HMC-1 cells (Figure 3).

### 2.3. The Effects of Atractylodin on PMA Plus A23187-Induced Multiple Molecular Targets Activation

**Figure 4:**
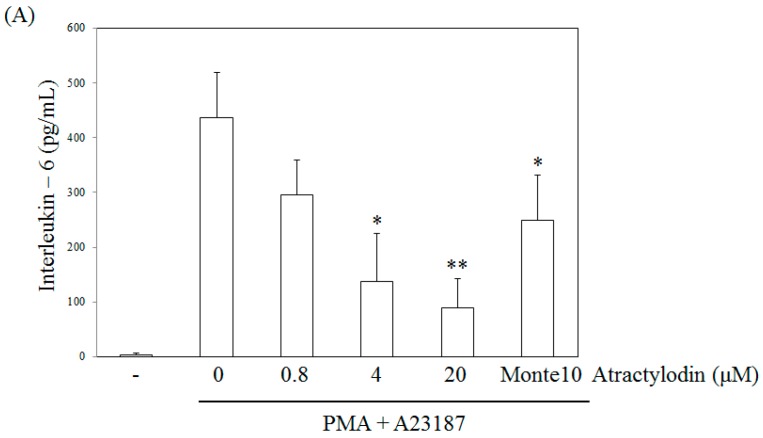

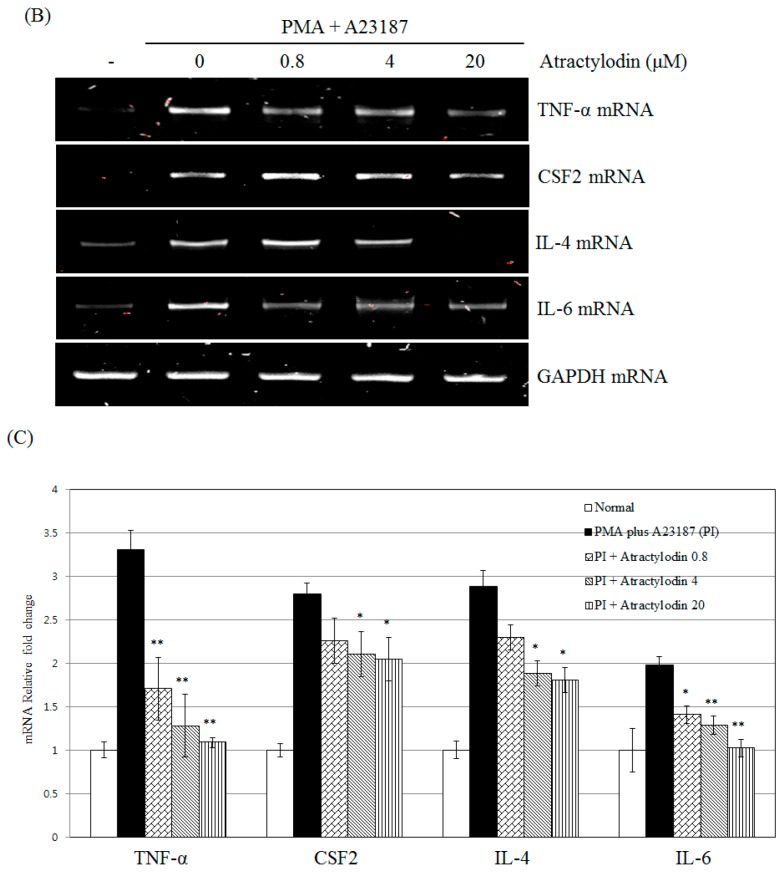
Effect of atractylodin on the release of IL-6 (**A**) and the expression of TNF-α, CSF2, IL-4, and IL-6 mRNA (**B**,**C**) in PMA plus A23187-stimulated HMC-1 and cell was treated with the indicated concentrations of atractylodin for 0.5 h prior to being incubated with PMA (50 nM) plus A23187 (1 μM) for 24 h and detected by ELISA, RT-PCR, and real-time RT-PCR, analysis as described in Materials and Methods. Monte10: montelukast 10 μM. Statistical significance: * *p* < 0.05 as compared to the PMA plus A23187 treated group. ** *p* < 0.005 as compared to the PMA plus A23187 treated group. Values shown are the mean ± S.E. of duplicate determinations from three separate experiments.

To evaluate the effects of atractylodin on the production of IL-6, we pretreated cells with this compound before stimulation for 24 h, and then analyzed the samples by ELISA. Atractylodin displayed an inhibitory effect on IL-6 production in stimulated HMC-1 cells with an IC50 value of 6.32 μM (Figure 4A). Montelukast was used as a positive control since it is a known inhibitor of mast cell activation [25]. A cell viability test following treatment of the human mast cells with these compounds revealed no toxicity up to 20 μM (data not shown). The effect of atractylodin on TNF-α, CSF2, IL-4, and IL-6 mRNA expression induced by PMA plus A23187 was examined using reverse transcription polymerase chain reaction (RT-PCR) and qRT-PCR. Treatment with PMA plus A23187 increased the mRNA expression of TNF-α, CSF2, IL-4 and IL-6, whereas atractylodin pretreatment suppressed this increase. Hence, atractylodin was found to potently inhibit TNF-α, CSF2, IL-4, and IL-6 production in the stimulated HMC-1 cells (Figure 4B,C).

### 2.4. PMA Plus A23187 Induced Phosphorylation of PLCγ1 and ERK is Mediated by NPM-ALK

**Figure 5:**
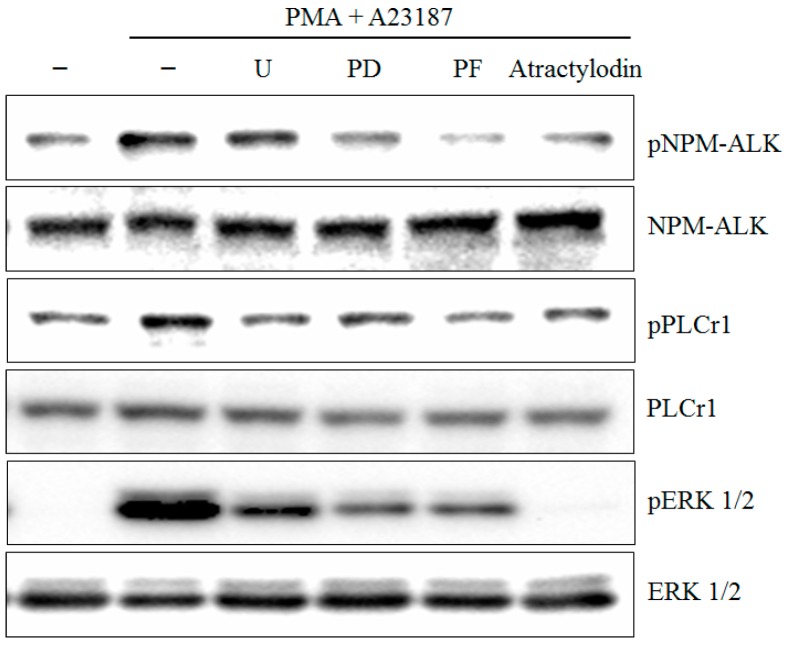
Effect of inhibitors on PMA plus A23187 induced NPM-ALK, PLC γ1, and ERK phosphorylation in HMC-1. HMC-1 were treated for 0.5 h in the presence or absence of 1 μM U73122 (U) or 1 μM PD98059 (PD), or 1 μM PF-02341066 (PF), or 4 μM atractylodin and then stimulated for 0.5 h with or without PMA plus A23187 in the continued presence or absence of inhibitors. Whole cell lysates were then analyzed by Western blot.

To test the role of signaling pathways on PLCγ1 and ERK activation in PMA plus A23187-induced human mast cell-1, specific inhibitors for 1 μM U73122 (U), 1 μM PD98059 (PD), and 1 μM PF-02341066 (PF), were treated to the cells. As shown in Figure 5, PF-02341066 inhibited PLCγ1 and ERK activation compared to that in stimulated cells. However, U73122 and PD98059 did not inhibited NPM-ALK activation.

### 2.5. The Effects of Atractylodin with Selective ALK Inhibitor on PMA Plus A23187-Induced Human Mast Cell-1

**Figure 6:**
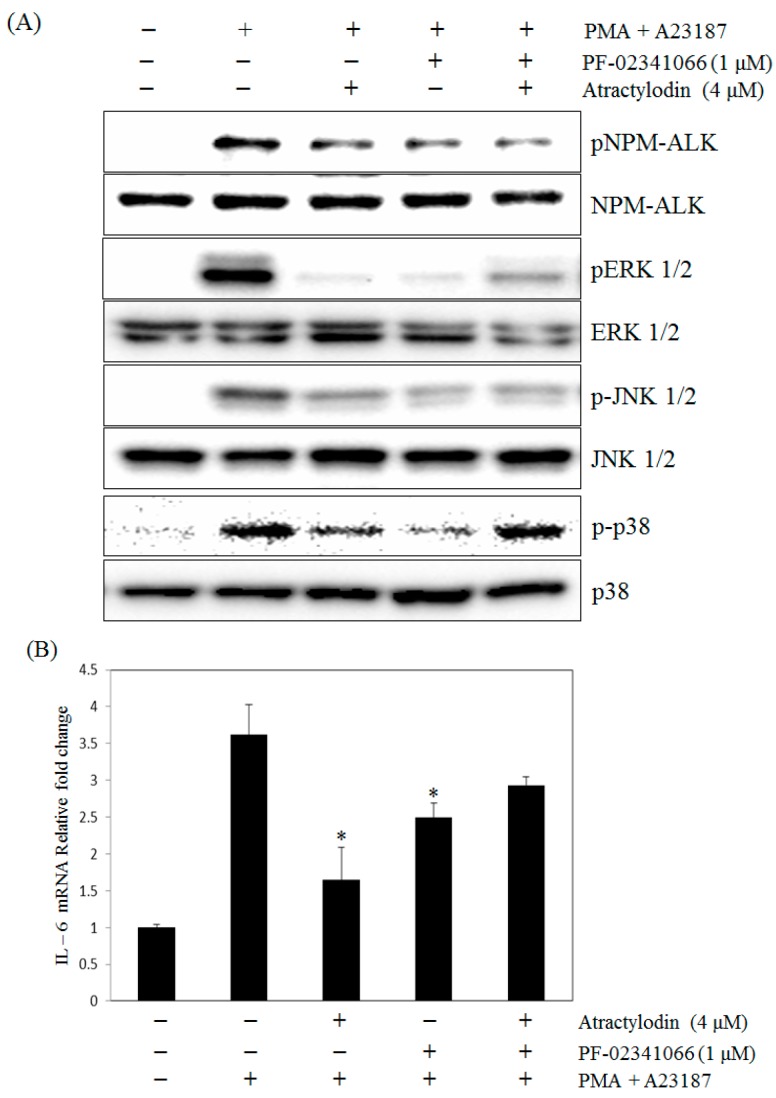
Effect of atractylodin and PF-02341066 on the phosphorylation of MAPKs (**A**) and the expression of IL-6 mRNA (**B**) in PMA plus A23187-stimulated HMC-1. (**A**) Cells were treated with or without atractylodin and PF-02341066 (1 μM) for 0.5 h and then treated with PMA plus A23187 for 0.5 h. Whole cell lysates were then analyzed by Western blot; (**B**) Cell was treated with atractylodin for 0.5 h prior to being incubated with PMA (50 nM) plus A23187 (1 μM) for 24 h and detected by real-time RT-PCR analysis as described in Materials and Methods. Statistical significance: * *p* < 0.05 as compared to the PMA plus A23187 treated group.

We next performed a series of experiments in which the function of ALK was abrogated using specific inhibitors. To compare the effects of atractylodin with PF-02341066 (Crizotinib), a potent inhibitor of ALK and IL-6 production, we measured the phosphorylation of ALK and the expression of IL-6 in HMC-1 cells. PF-02341066 suppressed the phosphorylation of ALK (Figure 6A) and also inhibited IL-6 mRNA expression in PMA plus A23187-stimulated HMC-1 cells. Furthermore, PF-02341066 decreased the inhibitory effect of atractylodin on IL-6 mRNA expression in stimulated HMC-1 cells (Figure 6B).

## 3. Discussion

Regulators of the ALK pathway, such as suppressors of cytokine production and protein inhibitors of activated ALK proteins, function to modulate the inflammatory immune response in an attempt to maintain homeostasis [26]. PLCγ is a downstream target of NPM-ALK, which contributes to its mitogenic activity [9]. Activated PLCγ cleaves the membrane-bound lipid, phosphatidylinositol-4,5-bisphosphate, into DAG, a stimulator of protein kinase C and inositol 1,4,5-trisphosphate, the ligand for the IP3 calcium channel receptor in the ER [27]. In addition, NPM-ALK exerts its activity through the activation of multiple signaling cascades that prompted us to investigate the role of the JAK2/STAT3 pathway [28]. Furthermore, it is reported that both PMA and A23187 are involved in the Janus family kinase (JAK)/signal transducer and activator of transcription (STAT) pathways [29]. NPM-ALK constitutively activates the AKT pathway, which is essential for NPM-ALK-mediated cell activation [30]. AKT is a multifunctional mediator of PI3-kinase activation in a variety of cell types [31]. Thus, JAK/STAT, AKT, and PLCγ seem to contribute to NPM-ALK activation, which plays a role in allergic inflammation. More specifically, ALK was involved in the activity of STAT, JAK, PLCγ, and AKT. Our study is the first molecular approach to demonstrate the function of ALK in mast cells. Several studies have identified the regulatory roles of ALK in lymphoma and immune systems. For example, regulation of anaplastic large T-cell lymphoma involves ALK, even though it is not always critical for immune responses [32]. Although the significance of the biological regulation of the ALK activation pathway is yet to be established, these lines of evidence highlight their importance in lymphoma and immunity.

ALK stimulation triggers activation of the MAPK pathway [33]. Additionally, MAPKs regulate the expression of pro-inflammatory genes [34]. It was previously reported that activation of HMC-1 cells by PMA plus A23187 is associated with phosphorylation of MAPKs [35]. In order to elucidate the mechanisms underlying the effects of atractylodin, we examined the possible effects of atractylodin on activation of MAPKs.

Cytokines play several roles in inflammatory responses, such as leukocyte proliferation and activation. Through the release of pro-inflammatory mediators, such TNF-α, CSF2, IL-4, and IL-6, mast cells are associated with the inflammation process [36]. The cytokines released from mast cells change the microenvironment, attracting neutrophils and basophils [37]. Therefore, inhibition of these pro-inflammatory cytokines is one of the key indicators of relived inflammatory disease. In this study, atractylodin suppressed the production of the inflammatory mediators, TNF-α, CSF2, IL-4, and IL-6, in HMC-1 cells induced by PMA plus A23187. These results indicate that the atractylodin in mast cells exerts anti-inflammatory effects by inhibiting both expression and secretion of pro-inflammatory cytokines.

Collectively, our findings confirm ALK as a positive regulator of mast cell activation, demonstrating a modulatory role of cytokines in the context of ALK signaling, thereby highlighting the ALK pathway as a potential drug target aimed at controlling mast cell response. Atractylodin is thought to exert its anti-inflammatory activity by inhibiting the release or expression of TNF-α, CSF2, IL-4, and IL-6 via suppressing ALK and MAPKs activation stimulated by PMA plus A23187 in human mast cells.

## 4. Materials and Methods

### 4.1. Cell Culture

A human mast cell line, HMC-1, was obtained from the Korea Research Institute of Bioscience and Biotechnology (Daejeon, Korea) and grown in Iscove's Modified Dulbecco’s Medium (IMDM) containing 10% fetal bovine serum and 100 U/mL penicillin/streptomycin sulfate. Cells were incubated in a humidified 5% CO_2_ atmosphere at 37 °C.

### 4.2. Drugs and Chemicals

IMDM, penicillin, and streptomycin were purchased from Hyclone (Logan, UT, USA). Bovine serum albumin, PMA, U73122, PD98059, PF-02341066, and A23187 were purchased from Sigma (St. Louis, MO, USA). Anti-mouse IL-6 antibody and biotinylated anti-mouse IL-6 antibody were purchased from BD Biosciences (BD Pharmingen, San Diego, CA, USA). p-STAT3, STAT3, p-JAK, JAK, p-PLCγ1, PLCγ1, p-AKT, AKT, p-ALK, ALK, β-actin, p-ERK, ERK, p-JNK, JNK, p-p38, and p38 antibodies were all purchased from Cell Signaling Technology, Inc. (Danvers, MA, USA). TNF-α, CSF2, IL-6, IL-6R, IL-8, and GAPDH oligonucleotide primers were purchased from Bioneer Corp. (Daejeon, Korea).

### 4.3. Extraction and Isolation

*Atractylodes lancea* was obtained from Daelim Korean Medical Market (Chungbuk, Korea) in February of 2013 and identified by Prof. Je-Hyun Lee from the College of Oriental Medicine, Dongguk University. A representative specimen (DGUH-20140001) has been deposited in the Medicinal Herb Garden of Dongguk University for reference purposes. *Atractylodes lancea* (942.11 g) was extracted with methanol at room temperature three times to obtain 402 g of solid extract (42.7% yield). The methanol extract (ALM) was suspended in H_2_O and then partitioned with n-hexane, chloroform, ethyl acetate, and n-butanol. The hexane-soluble extract (ALH, 53.34 g) was subjected to silica gel column chromatography using gradient mixtures of hexane:ethyl acetate (50:1 to 0.5:1) and 22 fractions (ALH1-ALH22) were collected. The ALH4 fraction (535.8 mg) was subjected to Sephadex LH-20 column chromatography and eluted with 100% methanol to produce 5 fractions (ALH4S1-ALH4S5). From the ALH4S4 fraction, one compound (atractylodin, 269.2 mg) was obtained. The structure of the compound was determined by its physico-chemical and spectral data (^1^H-NMR and ^13^C-NMR), which was in agreement with the structure reported in the literature [36].

### 4.4. Determination of Interleukin-6 Levels

Cells were seeded at 1 × 10^6^ cells/ml per well in 24-well tissue culture plates and pretreated with the indicated concentrations of compounds for 0.5 h before stimulation. After 24 h, the supernatant was decanted into a new micro-centrifuge tube, and the amount of interleukin-6 (IL-6) was determined using an ELISA kit according to the procedure described by the manufacturer (BD Bioscience). All assay steps were performed at room temperature, and all standards and samples were assayed in duplicate.

### 4.5. Immunoblot Analysis

Protein expression was assessed by Western blot analysis according to standard procedures. Briefly, HMC-1 cells were cultured in 60-mm culture dishes (2 × 10^6^ cells/mL) and then pretreated with various concentrations of atractylodin (0.8, 4, and 20 μM). After 0.5 h of pretreatment, PMA plus A23187 were added to the culture medium, and the cells were incubated at 37 °C for 20 min. Following incubation, the cells were washed twice in ice cold PBS (pH 7.4). The cell pellets were then resuspended in lysis buffer on ice for 15 min, after which the cell debris was removed by centrifugation. Protein concentration was then determined using BIO-RAD protein assay reagent according to the manufacturer’s instructions. Protein (20–30 μg of whole cell) was mixed 1:1 with 2× sample buffer (20% glycerol, 4% SDS, 10% 2-ME, 0.05% bromophenol blue, and 1.25 M Tris (pH 6.8)), loaded onto an 8 or 15% SDS-PAGE gel, and run at 150 V for 90 min. Cell proteins were transferred onto an ImmunoBlot polyvinylidene difluoride membrane (Bio-Rad) using a Bio-Rad semi-dry transfer system (Bio-Rad, Hercules, CA, USA) according to the manufacturer’s instructions. The polyvinylidene difluoride membrane was then incubated with primary Ab (diluted 1:500–1:1000) in 5% milk in Tris-buffered saline containing 0.1% Tween 20 overnight. The blots were washed three times with Tris-buffered saline (0.1% Tween 20) and incubated for 1 h with HRP-conjugated secondary anti-IgG Ab (diluted 1:2000–1:20,000). The blots were washed again three times with Tris-buffered saline (0.1% Tween 20), and immunoreactive bands were developed using the chemiluminescent substrate ECL Plus (Amersham Biosciences, Piscataway, NJ, USA).

### 4.6. Reverse Transcription Polymerase Chain Reaction (RT-PCR)

Total cellular RNA was isolated using a Trizol RNA extraction kit according to the manufacturer’s instructions. PCR products were electrophoresed on a 1.5% agarose gel and stained with ethidium bromide. The following specific primer sets were used (5′ to 3′):

IL-6: AAAGAGGCACTGGCAGAAAA (forward), AAAGCTGCGCAGAATGAGAT (reverse); GAPDH: GTCAGTGGTGGACCTGACCT (forward), AGGGGAGATTCAGTGTGGTG (reverse); TNF-α: AGCCCATGTTGTAGCAAACC (forward), GGAAGACCCCTCCCAGATAG (reverse); CSF2: TCTCAGAAATGTTTGACCTCCA (forward), AGGGCAGTGCTGCTTGTAGT (reverse); IL-4: ACTGCTTCCCCCTCTGTTCT (forward), CTCTGGTTGGCTTCCTTCAC (reverse). Gene-specific primers were custom-synthesized by Bioneer (Daejeon, Korea).

### 4.7. Quantitative Real-Time RT-PCR

Total cellular RNA was isolated using a Trizol RNA extraction kit according to the manufacturer’s instructions. Briefly, total RNA (1 μg) was converted to cDNA by treatment with 200 units of reverse transcriptase and 500 ng of oligo-dT primer in 50 mM Tris-HCl (pH 8.3), 75 mM KCl, 3 mM MgCl_2_, 10 mM DTT, and 1 mM dNTPs at 42 °C for 1 h. The reaction was then stopped by incubating the solution at 70 °C for 15 min, after which 1 µL of the cDNA mixture was used for enzymatic amplification. PCR reactions were performed using 1 μL cDNA and 9 μL master mix containing iQ SYBR Green Supermix (Bio-Rad), 5 pmol of forward primer, and 5 pmol of reverse primer, in a CFX384 Real-Time PCR Detection System (Bio-Rad) as follows: 3 min at 95 °C followed by 40 cycles of 10 s at 95 °C and 30 s at 55 °C, followed by plate reading. The fluorescence signal generated with SYBR Green I DNA dye was measured during the annealing steps. The specificity of the amplification was confirmed using a melting curve analysis. Data were collected and recorded by CFX Manager Software (Bio-Rad) and expressed as a function of the threshold cycle (CT). The relative quantity of the gene of interest was then normalized to the relative quantity of hypoxanthine phosphoribosyltransferase (ΔΔCT). The mRNA abundance in the sample was calculated by the equation 2^−(ΔΔCT)^. The following specific primer sets were used (5′ to 3′): IL-6: CCACACAGACAGCCACTCAC (forward), TGATTTTCACCAGGCAAGTCT (reverse); GAPDH: GAAGGTGAAGGTCGGAGTCA (forward), AATGAAGGGGTCATTGATGG (reverse); IL4: TGAACAGCCTCACAGAGCAG (forward), CTTGGAGGCAGCAAAGATGT (reverse); TNF-α: TCAGCCTCTTCTCCTTCCTG (forward), GCCAGAGGGCTGATTAGAGA (reverse); CSF2: TCTCAGAAATGTTTGACCTCCA (forward), AGGGCAGTGCTGCTTGTAGT (reverse). Gene-specific primers were custom-synthesized by Bioneer (Daejeon, Korea).

### 4.8. Statistical Analysis

Data from the experiments are presented as the mean ± S.E.M. The level of statistical significance was determined by analysis of variance (ANOVA) followed by Dunnett’s t-test for multiple comparisons. P values less than 0.05 were considered significant.
