# Correction: Chae, H.S.; et al. Atractylodin Inhibits Interleukin-6 by Blocking NPM-ALK Activation and MAPKs in HMC-1. *Molecules* 2016, 21, 1169

**DOI:** 10.3390/molecules21101412

**Published:** 2016-10-25

**Authors:** Hee-Sung Chae, Young-Mi Kim, Young-Won Chin

**Affiliations:** College of Pharmacy, Dongguk University-Seoul, 32 Dongguk-lo, Ilsandong-gu, Goyang-si, Gyeonggi-do 410-820, Korea; chaeheesung83@gmail.com (H.-S.C.); 0210121@hanmail.net (Y.-M.K.)

The authors wish to make the following correction to their paper [1]. In Panel D of Figure 2, the data was incorrectly displayed. The correct version of Figure 2D is as follows:

The change does not affect the scientific results. The manuscript will be updated and the original will remain online on the article webpage. The authors would like to apologize for any inconvenience caused to readers by these changes.

## Figures and Tables

**Figure 2:**
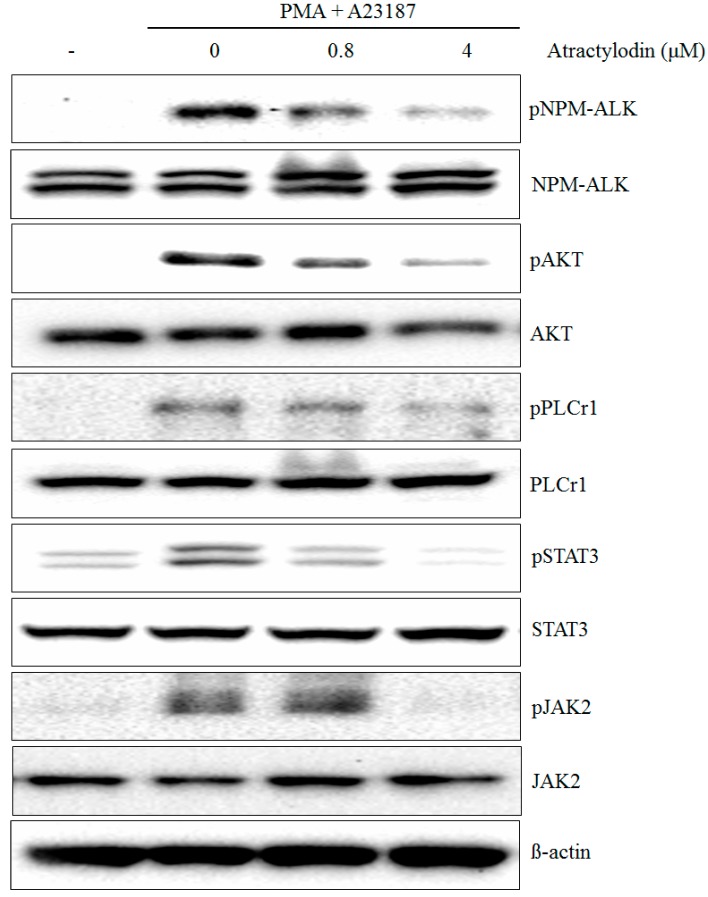
(**D**) HMC-1 was treated with the indicated concentrations of Atractylodin for 0.5 h prior to being incubated with PMA (50 nM) plus A23187 (1 μM) for 0.5 h and phospho and total NPM-ALK, AKT, PLCγ1, JAK2, and STAT3 were detected by immunoblot analysis as described in Materials and Methods.

## References

[1] Chae H.S., Kim Y.M., Chin Y.W. (2016). Atractylodin Inhibits Interleukin-6 by Blocking NPM-ALK Activation and MAPKs in HMC-1. Molecules.

